# An Amphiphilic Pyridinoyl-hydrazone Probe for Colorimetric and Fluorescence pH Sensing

**DOI:** 10.3390/molecules24213833

**Published:** 2019-10-24

**Authors:** Rosita Diana, Barbara Panunzi, Angela Tuzi, Stefano Piotto, Simona Concilio, Ugo Caruso

**Affiliations:** 1 Department of Agriculture, University of Napoli Federico II, via Università 100, 80055 Portici NA, Italy; rosita.diana@unina.it; 2 Department of Chemical Sciences, University of Napoli Federico II, via Cintia, 80126 Napoli, Italy; angela.tuzi@unina.it (A.T.); ugo.caruso@unina.it (U.C.); 3 Department of Pharmacy, University of Salerno, via Giovanni Paolo II 132, 84084 Fisciano SA, Italy; piotto@unisa.it; 4 Department of Industrial Engineering, University of Salerno, via Giovanni Paolo II 132, 84084 Fisciano SA, Italy; sconcilio@unisa.it

**Keywords:** pH sensor, fluorophore/chromophore probe, molecular dynamic simulation

## Abstract

A new pH sensor based on a substituted aroylhydrazide with a flexible side chain and a terminal trimethyl ammonium group (PHA^+^) was designed and synthesized. The terminal quaternary ammonium guarantees excellent solubility in water. At the same time, the probe is very soluble in hydrophobic envirornments. The pyridinoyl-hydrazone moiety acts as the pH-sensitive fluorophore/chromophore probe. Extensive physicochemical characterization has been performed on the bromide salt PHABr. DFT calculations, based on single-crystal X-ray data, permitted to rationalize the optical behavior. Molecular dynamics simulations permitted to clarify the mode of interaction with lipid membrane. The ability of the probe to change color and fluorescence in response to different pH and media of different polarity has been investigated. PHABr shows a remarkable pH-dependent behavior in both absorption and fluorescence spectra with high sensitivity and strong on-off switch effect at neutral pH, perceptible even to the naked eye.

## 1. Introduction

Cell functions are significantly affected by variation in the normal physiological pH conditions [1,2,3,4,5,6,7]. The accurate determination of pH is essential for the understanding of the chemical, biological and physical processes in the living organisms. Owing to the importance of pH measurement in the different and complex matrices of the biological systems, researchers developed a wide range of analytical methods for pH monitoring. The approach can be categorized in electrochemical or non-electrochemical methods [8]. Within non-electrochemical methods, optical response is a common strategy. Most of the well-established optical methods for pH detection are based on sensors able to respond to pH by a colorimetric and/or fluorescence change in water [9,10]. Sensory technology based on water-soluble molecules has attracted considerable interest [11,12,13]. Although in some applications pH electrode is irreplaceable, numerous chemosensors for biologically relevant targets in aqueous systems have been developed [9]. An important target is the lysosome, which is distinct from other cellular organelles because of its low pH [14]. Normally, cell membranes are monitored using fluorescent probes capable of recognizing different lipidic surrounds. Great efforts have been made to design fluorophores with high emission differences depending on the physical state of the membrane domain in which they are immersed [15]. For these reasons it is very important to have probes capable of discriminating between membranes of different composition, different pH and with a marked difference in emission. More importantly, metal ions such as iron, copper and zinc are recycled during autophagy into lysosomes or taken from the outside. Zinc and copper ions can be bound to the lysosomal metallothionein from which they are released during acidification [16].

In complex biological systems, where the pH of different organelles is strictly regulated, the measurement of pH is a crucial goal. Most fluorescence/colorimetric pH probes are limited because their solubility in water and in organic phase is not sufficient for most applications. Molecules possessing both hydrophilic and hydrophobic character can be employed in the biological aqueous and non-aqueous substrates [17,18]. Recently, efforts in the production of fluorescent/colorimetric pH sensors with an amphiphilic pattern have been extensively promoted as tools for biomedical purposes thanks to their secure cellular delivery and their high biocompatibility [18,19]. Among others, polymeric micelles, quantum dots, hydrogels, and nanoparticles [10,20,21,22] have been used.

In chemosensor-based pH detection, the probe molecules have a chromophore/fluorophore capable of existing in different tautomeric forms depending on pH. Numerous Schiff base chemosensors with sensing mechanism based on the excited state intramolecular proton transfer (ESIPT) are reported in scientific literature [9,23,24,25]. The inhibition of this intramolecular transfer is responsible for colour/fluorescence switch of the probe molecule. Aryl and aroyl hydrazone structures formally contain both the Schiff base moiety and an amidic fragment [9,26,27,28]. These probes, known as multitopic ligands for metal-organic architectures [29] and as sensors for cations, anions, neutral molecules [30,31,32,33], undergo keto-enol tautomeric interconversion. The equilibrium between the different forms with distinct properties can be influenced by solvent polarity and pH value. In particular, a non-coloured or weakly fluorescent, or coloured or fluorescent molecule, can be obtained depending on pH value or pH range (on-off switches) [34,35].

It is important to note that poor solubility and processability in water buffers generally limit the use of this kind of sensors. Recently, we designed and synthesized a series of organic molecules based on an aroyl-hydrazide core [32,33,36,37]. We also checked the effect of the insertion of a pyridinoyl moiety [36,38,39,40] on the spectroscopic response. It has been proved that N-heterocyclic derivatives can enhance the sensitivity and the rate of the tautomeric process involving pH determination [41].

**Scheme 1:**
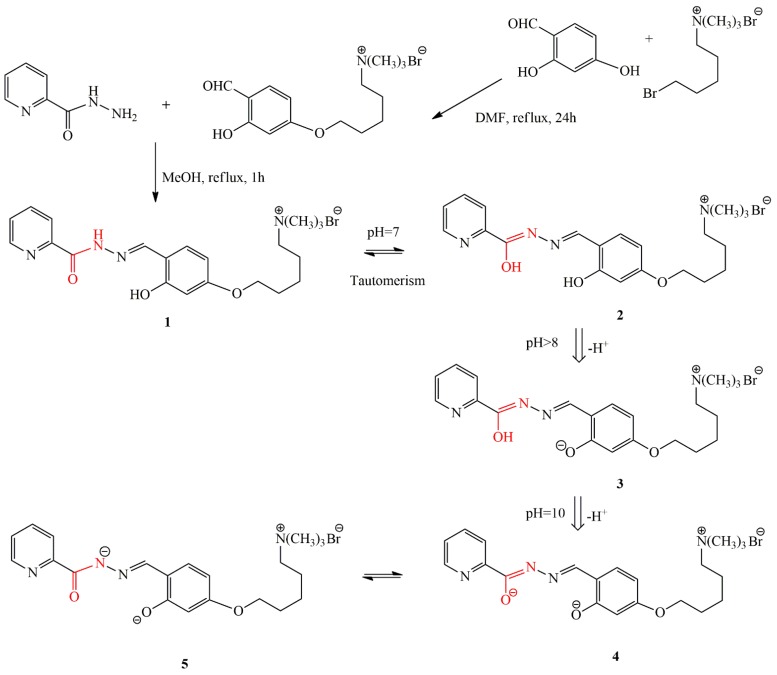
Synthetic procedure for probe PHABr and possible tautomeric forms 1–5 (involving the bonds marked in red) depending on pH.

Combining our experience in the synthesis of poly-functional ligands with our interest on chemosensors, we present a novel pH sensor in this paper. 5-(3-hydroxy-4-((2-picolinoylhydrazono)methyl)phenoxy)-N,N,N-trimethylpentan-1-aminium bromide (PHABr, see Scheme 1) is based on a substituted aroylhydrazide skeleton with a flexible five methylene side chain bearing a trimethylammonium bromide group. The charged amino group has been incorporated in the side chain of the fluorophore to achieve excellent water solubility. At the same time, solubility in the organic phase is sufficiently preserved [42,43].

Single-crystal X-ray analysis gave information about the structure of PHABr and was employed as a base for Density Functional Theory (DFT) calculations, used to rationalize the spectroscopic response. Chromogenic and fluorogenic pH sensing ability was investigated both in water and in organic solvents. By varying the pH, we checked the effects on the emission/absorption wavelength and intensity in solutions and in solid phase. Proton titration experiments in organic solvents and in water were also performed. PHABr exhibits remarkable pH-dependent behaviour both in absorption and in emission spectra, high sensitivity and sharp on-off switch effect at neutral pH. The cationic moiety PHA^+^ was used in the theoretical simulations. Despite its charged form, it can interact with the membrane. Owing to its ability to respond to pH variations, it is potentially able to discriminate membranes of the different composition both prokaryote and eukaryote. Amphiphilic nature of a probe is necessary but not sufficient condition to permit its use as a bilayer marker [44]. An ideal membrane probe is capable of inserting into the bilayer with a minimum perturbation of its physical state. Molecular Dynamics (MD) simulations of PHA^+^/POPC (Palmitoyl oleyl phosphocholine) system at different pH in water permitted to verify the effects on the structural parameters of the bilayer.

## 2. Results and Discussion

### 2.1. Synthesis and Tautomerism of Probe

Probe PHABr was obtained as summarized in Scheme 1, by condensation of a stoichiometric amount of 2-pyridinecarbohydrazide (or picolinohydrazide) and 5-(4-formyl-3-hydroxyphenoxy)-N,N,N-trimethylpentan-1-aminium bromide in methanol. PHABr undergoes keto-enol tautomeric interconversion. Tautomer 1 ketoamine is normally the most stable form in the solid state (as confirmed by X-ray analysis) and in a neutral solution., where it is stabilized by intermolecular or solute-solvent hydrogen bonds [45,46,47]. By increasing the pH, the possible deprotonated forms are 3, where the most acidic proton is lost, and finally 4 and its tautomer 5. This hypothesis is supported by the DFT computational study.

A full characterization of the ionic formyl precursor and the probe have been performed by NMR spectroscopy, elemental analysis, mass spectrometry measurements and DSC/TGA methods. Absorption and emission behaviour depending on the solvent and pH value are discussed below.

### 2.2. Photophysical Properties and pH Titrations

PHABr is highly stable in water and soluble up to 10 mM at physiological pH window. It can also be dissolved in common polar organic solvents such as chloroform, dioxane, acetone, ethanol, acetonitrile, DMF, DMSO, in concentration from 20 to 50 mM. Solubility increases with polarity and with the protic nature of the solvent. In less polar organic solvents, such as dichloromethane or THF, the solubility decreases to 10–20 mM. In biosensors, this amphiphilic nature is the base of efficient delivery into specific intracellular compartment of living cells [19].

UV-Vis and fluorescence spectra were recorded both in water and in ethanol or acetone solutions. These two organic solvents were selected for the miscibility with water and with Britton-Robinson buffer 1.00 M, prepared as described in the literature [48] and used in most of our experiments. Moreover, ethanol and acetone are a protic and an aprotic solvent with very similar polarity index (5.2 and 5.1, respectively). Compound PHABr shows similar photophysical behaviour in both of the solvents.

**Figure 1:**
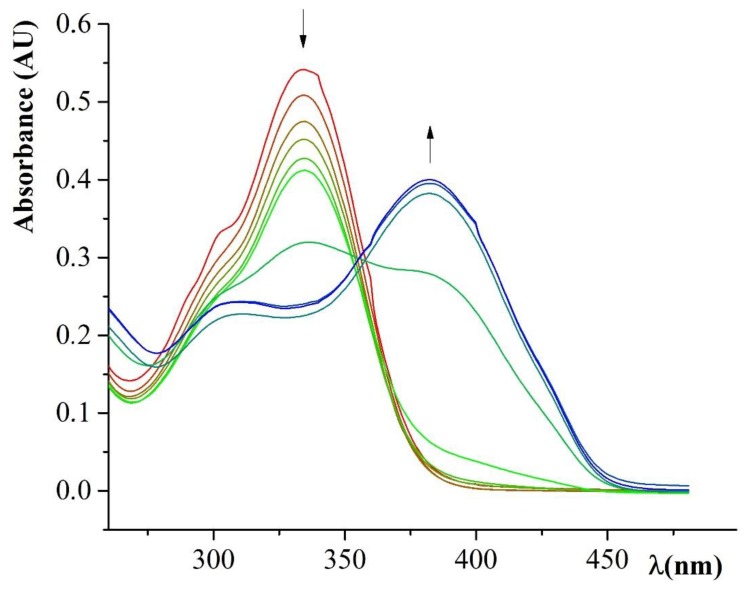
UV-Visible spectral titration of PHABr 100 μM in water from pH = 2.0 to pH = 11.0 using Britton-Robinson buffer. Color of the curves from red to green with increasing pH in acid solution and from green to blue with increasing pH in basic solution.

UV-Vis spectra of the hydrazide sensor PHABr have been recorded both in an organic solvent and in water, in 100 μM solutions using Britton-Robinson buffer at room temperature in the pH range 2.0–11.0. In the titration in water (Figure 1), the absorption band at 335 nm recorded starting from 2.0 pH value gradually decrease. This signal becomes a very broad band at neutral pH while a new band centred at 382 nm slowly grows up from 8.0 to 11.0 pH value. The red shift in the absorption titration pattern may correspond to the formation of 3 form, due to the gradual deprotonation of 1–2 tautomeric forms, until the fully deprotonation to 4–5 forms. The part of the broad absorption bands recorded after 390 nm is responsible for the yellow colour of the basic solutions.

**Figure 2:**
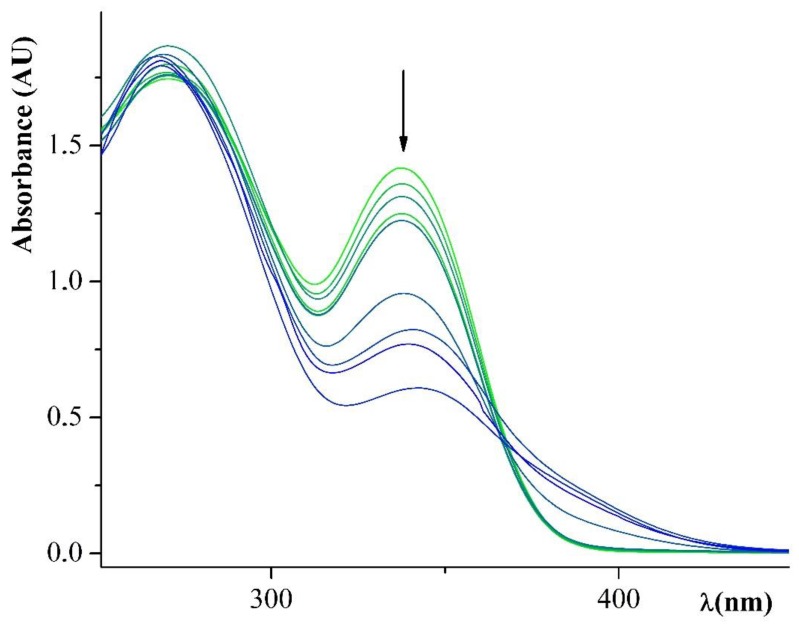
UV–Visible spectral titration of PHABr 100 μM in ethanol from pH = 2.0 to pH = 11.0 using Britton-Robinson buffer. Color of the curves from green to blue with increasing pH.

In the UV-Vis titration in ethanol (Figure 2) or in acetone, one can see a band at 341 nm whose intensity gradually decreases and widens with an increase in pH.

In this case, when the pH changes, there is no change in the maximum absorption. The yellow colour observed in alkaline solutions is due to the widening of the signal with a consequent increase in intensity in the red region. The absorbance molar coefficient calculated in water and ethanol at pH = 7.0 are in the same range: 56,550 and 63,400 M^−1^cm^−1^, respectively.

**Figure 3:**
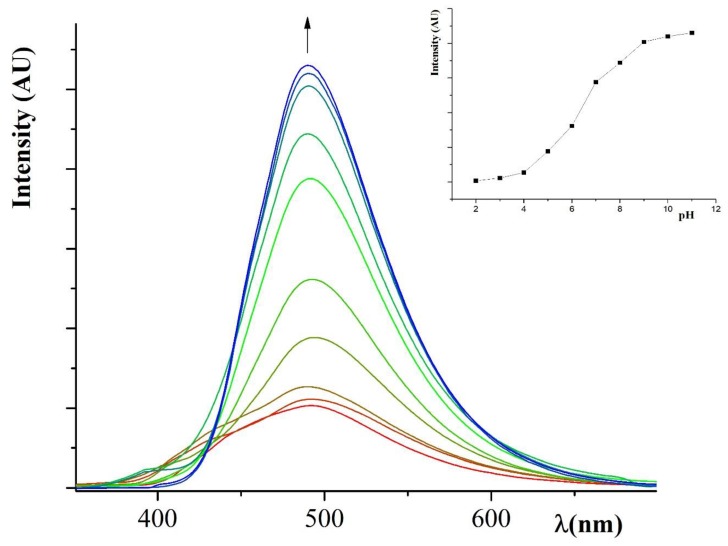
Fluorescence spectral titration of PHABr 20 μM in water from pH = 2.0 to pH = 11.0 using Britton-Robinson buffers, excitation wavelength 370 nm. Color of the curves from red to green with increasing pH in acid solution and from green to blue with increasing pH in basic solution.

**Figure 4:**
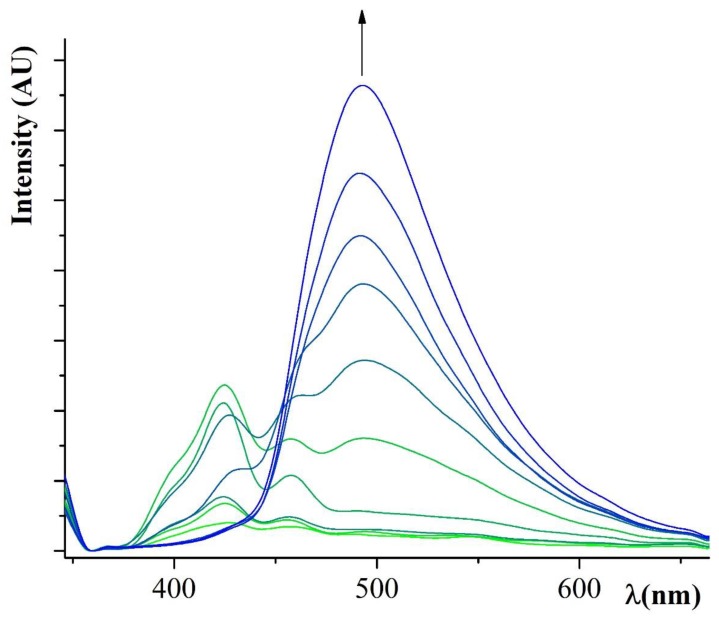
Fluorescence spectral titration of PHABr 20 μM in ethanol from pH = 2.0 to pH = 11.0 using Britton-Robinson buffers, excitation wavelength 340 nm. Color of the curves from green to blue with increasing pH.

Figure 3 and Figure 4 show the fluorescence titration diagrams using 20 μM PHABr solutions in water and in ethanol, respectively. In the water titration it can be seen how the band at 492 nm increases in intensity and is thinned, proceeding from pH 2 to pH 11. By plotting fluorescence intensity vs. pH, a sigmoidal pattern was observed (see inset in Figure 3) with flex at pH = 7.0.

Respect to titration in water, in ethanol (Figure 4) or in acetone a more elaborate pattern has been observed. An intense emission band peaked at 492 nm was recorded in basic solutions. Decreasing pH, the emission band gradually decreases in intensity and moves to 424 nm. The fluorescence mostly quenches at pH = 2.0.

A lot of reported pH probes have some drawbacks such as poor chemo- and photostability, scarce reversibility, and narrow Stokes shifts [49]. Probe PHABr Stokes shifts calculated in the basic range are 102 and 151 nm, respectively, for the aqueous and the ethanolic solution. Even larger values were calculated in acidic solution—157 and 183 nm respectively.

In the fluorescence/absorbance experiments, the signal was repeatedly turned on and off by alternately adding base (NaOH solution) or acid (HCl solution) to the probe. In all cases, the system gave a real-time response replicable and reversible, supporting the stability of the probe. Based on these characteristics, PHABr can technically be employed as an on-off colorimetric and fluorescence real-time pH sensor.

The fluorescence quantum yield (Φ) of PHABr was measured in solution at pH = 10.0 by relative methods using quinine sulfate QS as standard (0.546 in H_2_SO_4_ 1.00 N, excited at 365 nm) according to [24,50,51]. The value Φ = 2.40% is comparable to that reported for similar amphiphilic pH sensing systems [43,52] and also for intracellular pH sensing molecules [53]. The weak solution emission efficiency is typical of AIE (aggregation-induced emission) active molecules [24,54,55], and is due to the free molecular rotation, which activates a nonradiative decay. Nevertheless, in concentrated solutions, the intensity of emission increases until it becomes perceptible to the naked eye (see Section 2.3).

### 2.3. Naked Eye Detection

**Figure 5:**
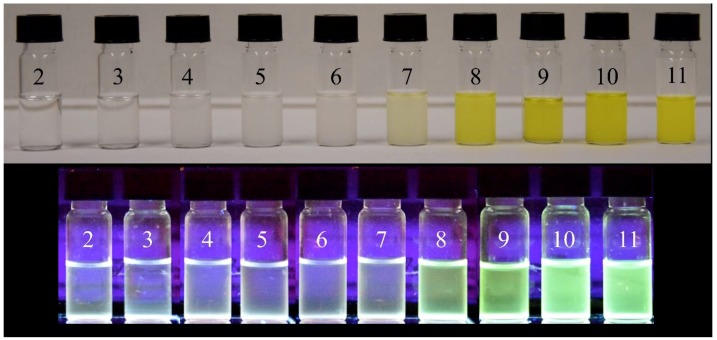
Color of 200 μM probe PHABr in buffer of pH 2.0, 3.0, 4.0, 5.0, 6.0, 7.0, 8.0, 9.0, 10.0, 11.0 photographed under visible light (light background) and UV lamp at 365 nm (dark background).

The probe PHABr shows a real-time naked-eye colorimetric response. Water solutions 200 μM of PHABr in 1.00 M Britton-Robinson buffer of pH 2.0, 3.0, 4.0, 5.0, 6.0, 7.0, 8.0, 9.0, 10.0, 11.0 were photographed under visible light and UV lamp at 365 nm (Figure 5). In natural light, PHABr is quite colourless up to pH = 7.0. The pale-yellow neutral solution is the first barely coloured solution, after that the colour turns to bright yellow in alkaline solutions (Figure 5, first line). A colorimetric response is observable in fluorescence as well. The same water solutions appear white up to pH = 7.0 and emit lime green under alkaline conditions, with a slight increase from 8.0–9.0 to 10.0-11.0. Interestingly, sensor PHABr turns in colour just at a neutral pH value. The sharp on-off switch effect both in natural and UV light makes it possible to identify acidic, neutral, and alkaline different regions unambiguously.

The fluorescence colorimetric response is observable under a UV lamp at 365 nm in organic media as well. PHABr scarcely emits in most of the organic solvents, and most of the solutions are colourless. In Figure S1 the probe has been photographed dissolved in distilled water (pH = 6.25, sample 8 of Figure S1) and in some common polar organic solvents at 100 μM concentration (samples 1–7). After addition of NaOH up to pH = 8.0, the solutions emit various shades of yellow-green colour. In the more polar solvents DMF, DMSO and water, the emission is more intense. In these solvents and ethanol and acetone, the fluorescence sharply turns-on from almost colourless in the pure solvent to lime green in the base-added solvent.

Contrary to similar hydrazone-based compounds with uncharged side chains [36], probe PHABr shows emission in the solid state (PLQY = 16% ± 1) as well. The emission is perceivable to the naked eye in the crystalline sample and when the probe is adsorbed on standard laboratory paper support (Figure S2). Since paper samples for everyday use (laboratory, domestic, industrial) are polluted by a small amount of calcium carbonate deriving from the production process, the probe appears pale yellow at acidic pH as well. However, the colour difference between the acid form and the basic form is clearly visible.

### 2.4. Selectivity of PHABr pH Sensor

**Figure 6:**
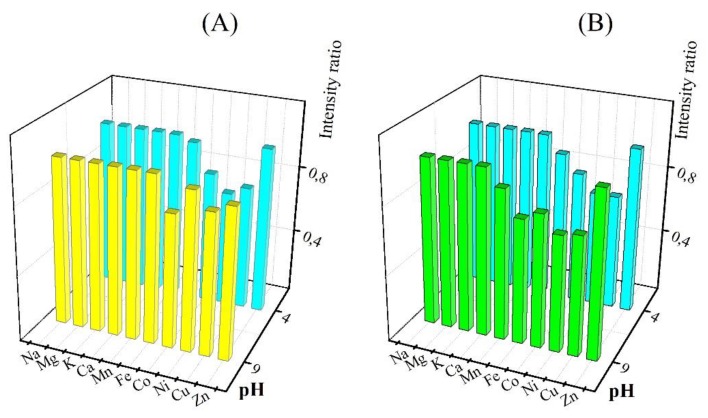
Metal selectivity profile of the sensor PHABr in bar representation. (**A**) change of absorbance maxima in buffer at pH = 4.0 (light blue) and pH = 9.0 (yellow) of PHABr in 20 μM water with a stoichiometric amount of cations. (**B**) change of emission maxima in buffer at pH = 4.0 (light blue) and pH = 9.0 (green) of PHABr in 100 μM water with a stoichiometric amount of cations.

Particular attention must be paid to interference due to some metal cations present in biological environments. Due to its potentially chelating nature, interference caused by metal cations in pH sensing of PHABr probe was expected. The selectivity of the probe over some metals was checked in water both at acidic and at basic pH values. Metals such as Na^+^, K^+^, Mg^2+^, and Ca^2+^ did not cause emission or absorption changes. On the contrary, most of the biologically relevant transition metal ions caused some change in PHABr response. In particular, Mn^2+^, Fe^3+^, Co^2+^, Ni^2+^, and Cu^2+^ cause a decrease in fluorescence emission in both pH conditions. The same metallic cations also slightly influence the absorption at acid and alkaline pH, as shown in Figure 6 at pH = 4.0 and pH = 9.0.

### 2.5. ^1^H NMR Spectral Study

**Figure 7:**
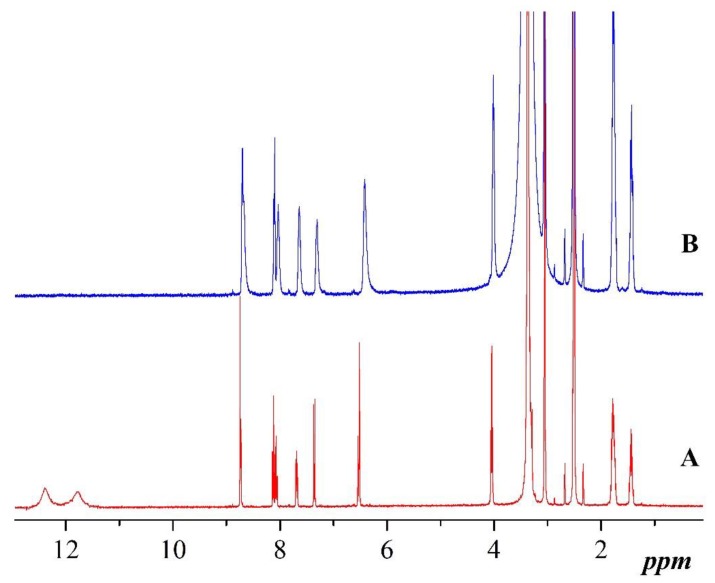
^1^H-NMR spectra of PHABr in DMSO-d_6_ in the absence (**A**) and in the presence (**B**) of NaOH.

^1^H NMR spectrum of the free compound shows a peak at 12.40 and 11.76 ppm, which indicates the presence of amidic and phenolic proton respectively. The peak at 8.75 ppm is related to the imine proton (shifted of 1.15 ppm respect to the same aldehydic proton of the formyl precursor, see Experimental section). Protons of methyl groups on charged nitrogens are visible as singlet at 3.05 ppm. Signals for O-CH_2_ appears as a triplet at 4.04 ppm and the other methylene groups at the lower field. Other aromatic protons appear in the region of 6.51–8.75 ppm. To investigate its structure in basic medium, ^1^H NMR spectrum of PHABr has been re-recorded after the addition of a minute amount of sodium hydroxide in DMSO-d_6_ (0.05mL of NaOH in D_2_O 1.00M were added to a solution of 12 mg PHABr in 0.70 mL of DMSO-d_6_,). There is some significant change in the spectrum in the presence of the base. In Figure 7B, the peaks at 12.15 and 11.80 ppm are absent, confirming the deprotonation of the probe in alkaline medium. The aromatic peaks undergo minimal shift confirming that in the presence of base the deprotonation of phenolic and of the alcoholic proton occurs with the formation of 4 form (in Scheme 1). The theoretical studies are in accordance with this experimental result.

### 2.6. X-ray Single Crystal Structure

**Figure 8:**
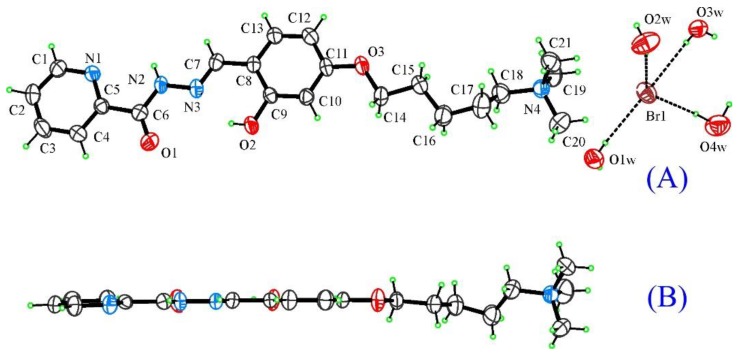
(**A**): Ortep view of PHABr normal to phenol ring with the cluster of H_2_O molecules around Br^-^ anion nearest to the N(trimethylammonium) atom. (**B**): view of the cationic moiety PHA^+^ down the edge of phenol ring. Thermal ellipsoids are drawn at 50% probability level, hydrogen bonds are drawn as dashed lines.

Single PHABr crystals suitable for the analysis of the crystalline X-ray structure were obtained by slow evaporation from an acetone/water solution at room temperature. Figure 8 shows the molecular structure of PHABr. In Supplementary Materials, we reported the crystal data and refinement details (Table S1), the most relevant bond lengths and angles (Table S2), and the hydrogen bonding geometry (Table S3).

**Figure 9:**
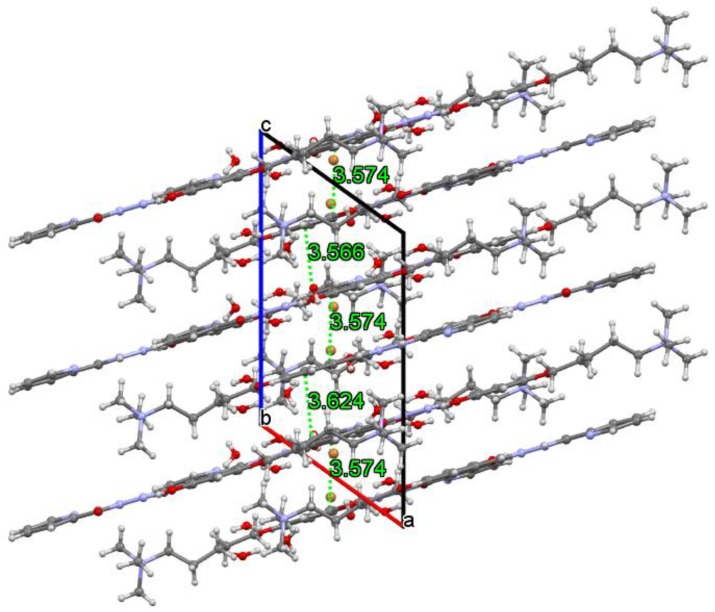
Crystal packing of PHABr viewed along b axis showing the molecular stacking at about 3.6 Å.

Compound PHABr crystallizes in the monoclinic P 2_1_/c space group with one cation, one anion and four crystallization water molecules contained in the asymmetric unit. The pattern of bond lengths and angles (Table S2) is in agreement with the more stable keto form 1 of Scheme 1. In particular, the C6-O1 bond distance (1.221(5) Å) is typical of a carbonyl double bond. The presence of the N(amide) hydrogen atom is unequivocally demonstrated by its localization in difference Fourier maps followed by the free refinement in the crystal structure analysis. In PHA^+^, the N(pyridine) and the N(amide) atoms are mutually cis disposed and the intramolecular hydrogen bond between the OH donor and the N(imine) acceptor groups favour a planar conformation of phenol ring (N3-C7-C8-C13 = −178.5(4)°; O2-H···N3 = 0.73(5), 1.97(6), 2.594(5) Å, <143(6)°]. The pyridine and the phenol groups are quite co-planar with a dihedral angle of 4.2(1)° between their mean planes. The shape of the PHA^+^ cation is essentially planar as the lateral alkyl-N (trimethyl ammonium) group also lies on the plane of the phenolic ring. (Figure 9). The crystal packing is dominated by electrostatic interactions and intermolecular hydrogen bonds among the strong donor/acceptor groups of PHA^+^ and the solvent water molecules of crystallization (Table S3). The OH and NH (amide) groups act as donors towards the solvent H2O molecules, which in turn form an H-bonded cluster around the Br^-^ anion. The pattern of anions and cations in the crystal packing is reported in Figure S3. The bromine anion is surrounded by a cluster of four H-bonded water molecules in the first coordination sphere and by three -N(CH_3_)_3_ groups in the second coordination sphere with a mean N4···Br1 distance of about 4.8 Å (Figure S3).

Layers of PHA^+^ molecular ions are arranged at a distance of about 3.6 Å (direction a–c) to maximize stacking π···π (Figure 9 and Figures S4–S5). The significant presence of π···π stacking interactions between aromatic rings in the crystal packing is confirmed (Figure S6) by of the curvedness and shape index Hirshfeld surfaces and by the two-dimensional fingerprint plot calculated using CrystalExplorer17.5 [56].

### 2.7. Computational Studies

**Figure 10:**
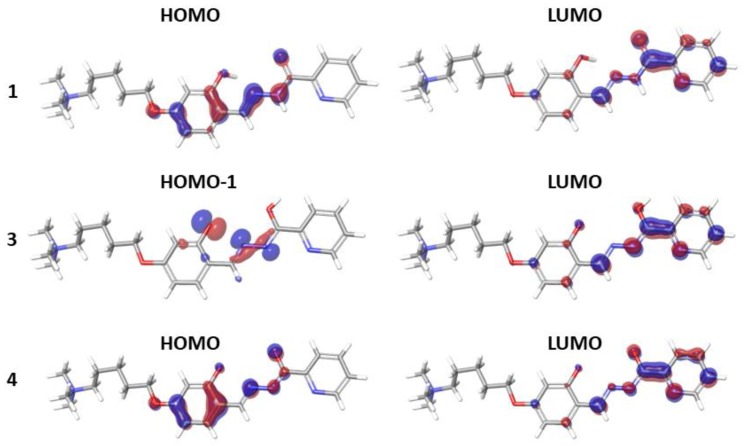
Frontier molecular orbital surfaces calculated at the B3LYP/6-31G(d,p) level for the most relevant forms (1, 3 and 4 of Scheme 1) of the probe without Br^−^ ion. Only the orbitals corresponding to the most intense transitions are shown.

The excitation energies were obtained at the density functional level by using the time-dependent perturbation theory approach (TDDFT) with the adiabatic local density approximation. It is highly reliable in obtaining accurate predictions for excitation energies and oscillator strengths. Calculations performed on the cation part of the probe PHA^+^ in water showed the localization of the most relevant frontier orbitals (see Figure 10).

We have calculated the main transitions for forms 1, 3, and 4 (see Scheme 1) of the probe without bromide ion. The main transition for form 1 corresponds to HOMO→LUMO, where the HOMO is mainly localized on the phenol ring and the LUMO on the pyridine ring. The main transition for form 4 is also HOMO→LUMO, but for the form 3 is HOMO-1→LUMO because of the odd number of electrons of form 3. As shown in Table 1, the absorption (λ_max_), and emission (E_max_) peaks in water are in excellent agreement with the experimental data. It is interesting to note that the oxidation potential of PHA^+^ is similar to that of very common fluorophores, such as the Nile red (+0.76V) or rhodamine B (+1.00 V). The value of the reduction potential, on the other hand, is significantly higher than those of Nile red and rhodamine B (−1.89 V and −1.80 V respectively). These results suggest further investigation to evaluate the possibilities of using PHA^+^ for the development of new redox potential sensors.

### 2.8. Molecular Dynamics and Analysis

**Figure 11:**
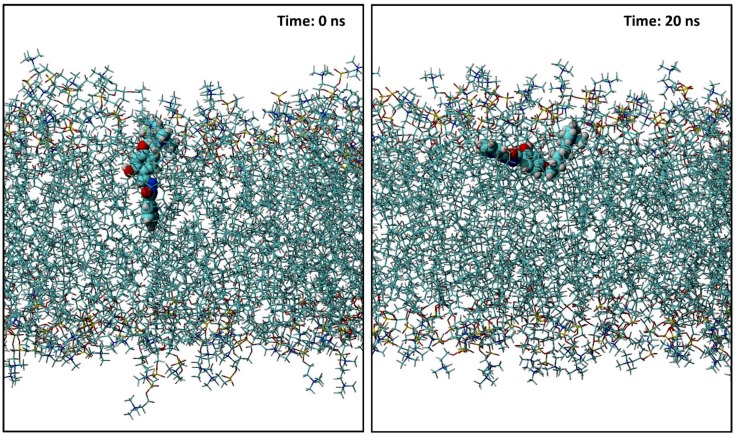
MD simulation snapshot after 20 ns of PHA^+^ probe interacting with a POPC membrane at pH = 7.0.

MD simulations can offer key information about the interactions of a molecule with a lipid bilayer. It can be used to explore binding, permeation and accumulation of relevant molecules in membranes and to monitor pH changes. We analysed the behaviour of PHA^+^ (cationic part of form 1, at pH = 7.0) and of the zwitterion PHA^±^ (form 3 without bromide ion, that is; PHA^+^ at pH = 9.0) molecular species within a model membrane of POPC, in simulation runs of 50 ns. The simulation confirmed the interaction of both molecules with the model membrane of POPC, as shown in Figure 11 for PHA^+^.

**Figure 12:**
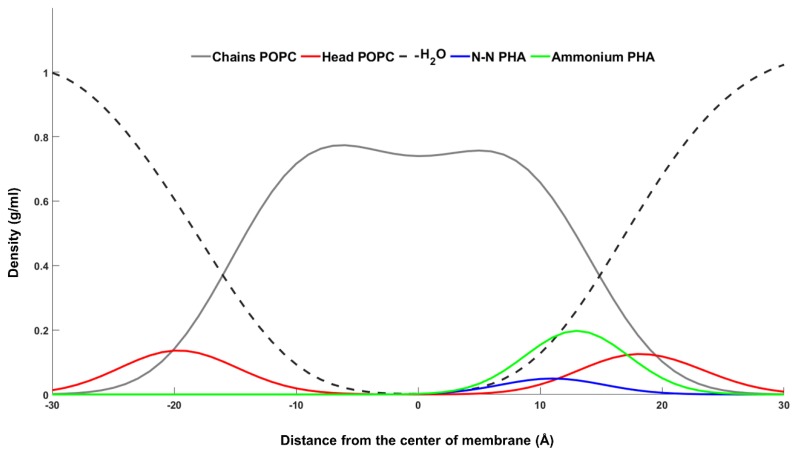
Density profile of the system PHA^+^/POPC at pH = 7.0. Dashed line: water molecules; grey line: carbon atoms of hydrocarbon chains of POPC; blue line: N-N fragment of the PHA^+^ molecule; green line: ammonium of PHA^+^ molecule; red line: head of POPC. For the sake of visualization, the peaks of PHA^+^ have been increased by 25 times.

Though bearing a net charge, the PHA^+^ ion is still capable of interacting with lipid membranes as revealed by the density profile, which indicates the distribution of the atoms along the membrane bilayer (Figure 12).

**Figure 13:**
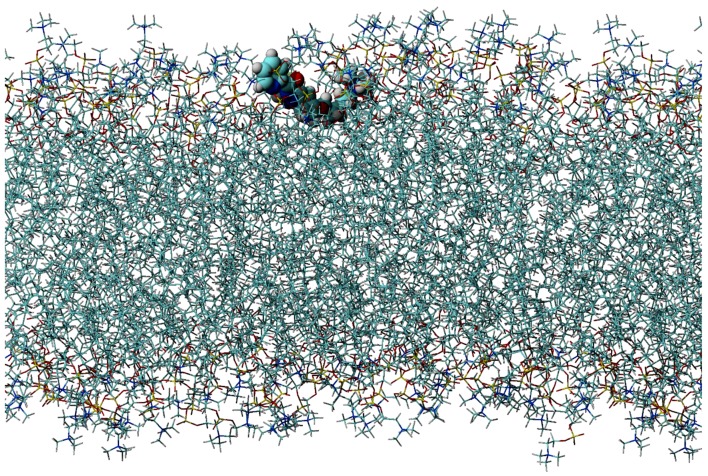
MD simulation snapshot after 20 ns of the PHA^±^ moiety interacting with a POPC membrane at pH = 9.0.

The blue and green lines represent, respectively, the N-N fragment and the ammonium group of PHA^+^. Their position confirms that the aromatic component and the charged nitrogen are inserted in the bilayer with the charge regions close to the POPC head and to the water. From Figure 13, it appears that the PHA^+^ molecule is positioned between the polar heads and the nonpolar tails of the POPC.

Interestingly, the change of pH and the subsequent change of net charge of the probe does not modify their interactions significantly with the membrane. PHA^+^ lies parallel to the membrane below the headgroup level, with only the positive charge capable to sporadically interact with the water bulk. PHA^±^ lies on the membrane with the negative charge capable of attracting the choline ammonium group and the positively charged nitrogen buried just below the phosphate moieties (see Figure 13).

The presence of a positive and a negative charge on the probe at pH = 9.0 prevents its insertion in the bilayer, and the tiny dimension of the probe permits an optimal interaction with the POPC charges. To verify the stability of the physical state of the membrane, we evaluated the thickness of the membrane with and without PHA^+^, and SCD (deuterium order parameter). Membrane thickness of the PHA^+^/POPC system is measured as the average distance between the centre-of-mass of the phosphorus atoms of opposite leaflets for the last 5 ns of simulation.

The calculated thickness was subsequently compared with the membrane thickness of pure POPC. The SCD profile of the lipid bilayer shows the variation of the order parameter with the position of the segment in the chain and is an expression of the average angular fluctuations around the normal bilayer. If the chains are in an all-trans conformation, the SCD value is close to 1. As shown in Figure S7b (palmitic), the saturated chain is not affected by the presence of PHA^+^ and PHA^±^ and the effect on the unsaturated chain (Figure S7a, oleic) reveals a bland change only. Summarizing, the presence of the probe in the pH range 7–9 does not affect the thickness of the membrane (Table 2), nor the lipid chain order (Figure S7). The POPC headgroup packing can be monitored by the area per lipid, as shown in Figure S8. At pH = 7.0, the presence of the charged PHA^+^ does not alter the head tiling. Interestingly, at pH = 9.0 the zwitterionic form PHA^±^ is responsible for slightly tighter packing of the lipids.

## 3. Experimental

### 3.1. General Remarks and Instrumentation

Optical observations were performed using a Zeiss Axioscop polarizing microscope (Carl Zeiss, Oberkochen, Germany) equipped with an FP90 Mettler heating stage (Mettler-Toledo International INC MTD, Columbus, Ohio, USA). Phase transition temperatures and enthalpies were measured using a DSC scanning calorimeter Perkin Elmer Pyris 7 (PerkinElmer, Inc., Waltham, MA, USA) at a scanning rate of 10 °C/min, under nitrogen flow. The structures of intermediates and complexes were confirmed by ^1^H NMR. The spectra were recorded in DMSO d_6_ using Bruker Spectrometers operating at 400 MHz. Mass spectrometry measurements were performed using a Q-TOF premier instrument (Waters, Milford, MA, USA) equipped by an electrospray ion source and a hybrid quadrupole-time of flight analyzer. Mass spectra were acquired in positive ion mode, in 50% CH_3_CN solution, over the range 100–1000 m/z. Instrument mass calibration was achieved by a separate injection of 1 mM NaI in 50% CH_3_CN. Data were processed using MassLynx software (Waters, Milford, MA, USA). UV-Visible and fluorescence spectra were recorded with JASCO spectrometers (JASCO Inc., Mary’s Court, Easton, MD, USA). The PLQY of films have been measured on quartz substrates by a Fluorolog 3 spectrofluorometer (Horiba Jobin Instruments SA, Kyoto, Japan), within an integrating sphere equipped with an optical fiber connection.

Compounds 2-pyridinecarbohydrazide (picolinohydrazide), (5-bromopentyl) trimethylammonium bromide and 2,4-dihydroxybenzaldehyde are commercially available products by Aldrich (Sigma-Aldrich Corporation, St. Louis, MO, USA).

### 3.2. Synthetic Procedures

Preparation of 5-(4-formyl-3-hydroxyphenoxy)-N,N,N-trimethylpentan-1-aminium bromide: the precursor was prepared by reaction of 2.00 g of (5-bromopentyl) trimethylammoniumbromide (6.92 mmol), 0.956 g of 2,4‑dihydroxybenzaldehyde (6.92 mmol) and 0.400 g of K_2_CO_3_ in 40 mL of acetonitrile. The reaction was kept under stirring at reflux for 24 h, under nitrogen atmosphere. After this time, the suspension was cooled and the solid removed by filtration. The solution was dried under vacuum and the precipitate crystallized from acetone/hexane. A whitish solid was obtained and dried at 60 °C. Yield: 65%. Mp = 67.22 °C, ΔH = 25.11 J/g. ^1^H NMR (400 MHz, DMSO-d6, 25 °C): δ = 10.00 (s, 1H), 7.62 (d, 1H), 6.58 (m, 1H), 6.51 (d, 1H), 4.07 (t, 2H), 3.36 (m, 2H), 3.07 (s, 9H), 1.77 (m, 4H), 1.42 (m, 2H) ppm. HRMS(ESI): *m/z* calculated for C_15_H_24_NO_3_^+^: 266.18; found 266.24 [M]^+^ (see Figure S9). Elemental analysis calculated (%) for C_15_H_24_NO_3_Br: C, 52.03; H, 6.99; N, 4.05; found: C, 52.63; H, 9.88; N, 4.11.

Preparation of PHABr: to 0.345 g (1.00 mmol) of 5-(4-formyl-3-hydroxyphenoxy)-N,N,N-trimethylpentan-1-aminium bromide dissolved in 15 mL of methanol 0.137 g (1.00 mmol) of picolinohydrazide were added. The reaction was kept under stirring at reflux for 30 min. After this time, the solution was cooled in an ice bath, with consequent precipitation of a pale yellow crystalline solid. The solid was recovered by filtration and crystallized from methanol. Yield: 80%. Mp = 272.00 °C, ΔH = 57.29 J/g. A solid-solid transition at 210.22 °C (with recrystallization from sheet to needle-shaped crystals) was detected and confirmed by optical observation. Decomposition temperature (calculated at 5% weight loss in N_2_) = 280 °C.

^1^H NMR (400 MHz, DMSO-d6, 25 °C): δ = 12.40 (s, 1H), 11.76 (s, 1H), 8.75 (m, 2H), 8.12 (m, 2H), 7.70 (d, 1H), 7.35 (d, 1H), 6.51 (d, 2H), 4.04 (t, 2H), 3.35 (m, 2H), 3.05 (s, 9H), 1.78 (m, 4H), 1.44 (m, 2H) ppm.

HRMS(ESI): *m/z* calculated for C_21_H_29_N_4_O_3_^+^: 385.22; found 385.18 [M]^+^ (see Figure S10).

Elemental analysis calculated (%) for C_21_H_29_N_4_O_3_Br: C, 54.20; H, 6.28; N, 12.04; found: C, 54.03; H, 6.33; N, 12.00.

### 3.3. UV-Visible and Fluorescence pH Titrations of PHABr

Stock solutions (1.00 M) of Britton-Robinson buffer (at 2.0, 3.0, 4.0, 5.0, 6.0, 7.0, 8.0, 9.0, 10.0, 11.0 pH value) prepared as described in literature [57] in bidistilled water (pH 6.25) were prepared. For fluorescence and absorbance titrations solutions of PHABr (100 and 20 μM, respectively) were prepared in bidistilled water, ethanol or acetone. Titration of the probe was performed by adding 60 μL of the buffer stock solutions to 2.5 mL of PHABr dissolved in the solvent. After mixing for a few seconds, the absorption or fluorescence spectra were recorded at room temperature.

### 3.4. Naked Eye Detection of PHABr at Different pH Values

For the naked eye detection shown in Figure 5, solutions 200 μM of PHABr in 1.00 M buffer of pH 2.0, 3.0, 4.0, 5.0, 6.0, 7.0, 8.0, 9.0, 10.0, 11.0 were prepared and immediately photographed. For Figure S1, PHABr was dissolved in chloroform, dioxane, acetone, ethanol, acetonitrile, DMF DMSO and distilled water (pH = 6.25) at 100 μM concentration before and after addition of a solution of NaOH at pH = 8.0 (NaOH dissolved in ethanol 0.1 M). The samples were immediately photographed. In Figure 6, a bi-crystallized sample of probe PHABr was employed in the Petri dish. The laboratory paper employed was Labor filter paper, 67 g/m^2^, minimum porosity. It has been soaked using a 0.01 M water solution of the probe and Britton-Robinson buffers 1.00 M at pH 2.0, 4.0, 7.0, 9.0 and 11.0.

### 3.5. Crystallographic Determination and Data

Single PHABr crystals suitable for the analysis of the crystalline X-ray structure were obtained by slow evaporation of acetone/water solution at room temperature. One selected crystal was mounted at ambient temperature on a Bruker-Nonius KappaCCD diffractometer (Bruker Corporation, Billerica, MA, USA) (graphite monochromated MoK_α_ radiation, λ = 0.71073 Å, CCD rotation images, thick slices, *φ* and *ω* scans to fill asymmetric unit). Semiempirical absorption corrections (SADABS [58]) were applied. The structure was solved by direct methods (SIR97 program [59]) and anisotropically refined by the full matrix least-squares method on F^2^ against all independent measured reflections using SHELXL-version 2018/3 [60] and WinGX software-version 2014.1 [61]. In PHABr crystallization, H_2_O is present. The H atoms bound to N(amide), to the hydroxy OH group and to the O(water) were located in difference Fourier maps and their coordinates freely refined (*U*_iso_(H) equal to 1.2 *U*_eq_ of the carrier atom). All the other hydrogen atoms were introduced in calculated positions and refined according to a riding model (C-H distances equal to 0.93–0.97 Å and *U*_iso_(H) equal to 1.2 *U*_eq_ of the carrier atom). Crystal data and structure refinement details are reported in Table S1. Selected bond lengths and angles are reported in Table S2. Hydrogen bonds geometry is reported in Table S3. The figures were generated using ORTEP-3 [62] and Mercury CSD 4.0 [63] programs.

Crystal data were deposited at Cambridge Crystallographic Data Centre with assigned number CCDC 1939629. These data can be obtained free of charge from www.ccdc.cam.ac.uk/data_request/cif [64].

### 3.6. Theoretical Calculations

All quantum calculations were performed at the DFT/B3LYP level of theory by using the programs Jaguar in Schrödinger Release 2017-4 [65]. Geometry optimizations were performed with the B3LYP functional and the LACVP** basis set. For the charge assignment, the NBO method was used. The energies of the optimized structures are re-evaluated by additional single-point calculations on each optimized geometry using Dunning’s correlation-consistent triple-ζ basis set cc-pVTZ (-f), which includes a double set of polarization functions. Vibrational frequency calculation results based on analytical second derivatives at the B3LYP/6-31G**(LACVP**) level of theory were used to confirm proper convergence to local minima and to derive the zero point- energy (ZPE) and entropy corrections at room temperature, where unscaled frequencies were used. The absorption values were calculated from vertical excitation energies computed with TD-DFT in the Tamm-Dancoff [66] approximation at the neutral molecule geometry. The solvent water was modeled by the Poisson Boltzmann Solver, PB [67]. “Scaled” HOMO and LUMO energies are calculated from the computed redox data using the following expressions:

Absolute_Electrode = NHE_Energy + Electrode_Potential

Orbital_Energy = Absolute_Electrode + Redox_Potential

where NHE_Energy is the energy of the NHE electrode in water, taken to be −4.28 V, and Electrode_Potential is the potential of the chosen electrode relative to NHE.

### 3.7. Molecular Dynamics

The MD simulations were performed with the software YASARA Structure 17.3.30 [68]. The AMBER14 force field was used, with long-ranged PME potential and a cut-off of 8.0 Å. POPC lipid bilayer was used as a model. Each monolayer of the membrane consisted of 69 lipids. A periodic simulation cell was used under NPT ensemble, coupling the system to a Berendsen thermostat and barostat combined with control of solvent density as implemented in the software YASARA-version 19.7.10. The charges were assigned at pH = 7.0, in which the probe bears a positive charge (PHA^+^), and at pH = 9.0, in which the cationic part of the probe is a zwitterion (PHA^±^). The simulation box was filled with water with a density of 0.997 g/mL. The simulation cell was neutralized with NaCl with a final concentration of 0.9%. After equilibration of 300 ps, the probe was incorporated into a membrane, placing the intercalated aromatic component in the membrane and the charged nitrogen towards the heads. Short energy minimization was performed to optimize membrane geometry and to fill the membrane pore around the probe. The MD simulation was then initiated at 298 K and integration time steps for intramolecular forces every 1.25 fs. Simulation snapshots were saved at regular time intervals of 250 ps. The simulation last 50 ns. The complete MD protocol is described in [69]. The deuterium order parameter (SCD) is a measure of the motional anisotropy of the particular C-D bond investigated and yields its time-averaged orientation and is defined as:

$$S_{CD}=\frac{1}{2}\left(3\bar{cos^{2}\Theta }-1\right),$$

where *Θ* denotes the instantaneous angle between the C-D bond and the direction of the bilayer normal.

## 4. Conclusions

PHABr probe shows a remarkable pH-dependent behaviour in both absorption and fluorescence spectra with high sensitivity and marked naked eye on-off switch effect at neutral pH. Thanks to its amphiphilic nature, PHA^+^ can potentially interact with the cell membrane discriminating different membrane compositions without significantly altering the bilayer organization. These observations suggest that PHA^+^ and its salts could find application in the field of membrane physical state sensors. In addition, the calculated reduction potential value suggests possible applications such as redox sensors. The high Stoke shift, the capability to interact with membranes, the sharp response to pH changes, and the solvatochromism, makes PHABr an excellent candidate to develop a new generation of biosensors. Its ability to interact with membranes of different composition and polarity, to emit according to pH and to respond to the presence of important ions such as those of iron and zinc, make PHABr an extremely promising probe for the study of cellular systems in vivo in general and lysosomal systems in particular.

## Figures and Tables

**Table 1:** Electro-optical properties calculated of PHA^+^ (as cationic part of form **1** in Scheme 1).

Oxidation Potential (eV)	Reduction Potential (eV)	λ_max_ (nm)	E_max_ (nm)	Scaled HOMO (eV)	Scaled LUMO (eV)	-LUMO-HOMO (eV)
0.99	−3.86	390	516	−5.52	−0.67	4.85

**Table 2:** Membrane thickness of pure POPC membrane and upon the insertion of the probe.

	POPC Membrane	PHA^+^/POPC at pH 7	PHA^±^/POPC at pH 9
**Membrane Thickness**	37.5 ± 0.3	37.7 ± 0.4	37.8 ± 0.1

## Supplementary Materials

The following are available online at https://www.mdpi.com/1420-3049/24/21/3833/s1, Figure S1: PHABr sensor in 100 μM solutions of different solvents, Figure S2: Colour of probe PHABr as a crystalline solid in natural light (Petri dish on the left) and resting on the UV lamp at 365 nm (the same Petri dish on the right). Colour of common laboratory paper soaked in the probe at pH 2.0, 4.0, 7.0, 9.0, and 11.0 photographed at visible light (above) and under UV lamp at 365 nm (down), Figure S3: Up: Br- anion surroundings with four H-bonded water molecules contained in the first coordination sphere and three -N(CH3)3 groups in the second coordination sphere at N4…Br1 distance of about 4.8 Å. Down: Pattern of anions and cations in the crystal packing. Water molecules and H atoms are not reported for clarity. Bromine and N(trimethylammonium) atoms are drawn as ball-and-stick stile, all the other atoms as wireframe style, Figure S4: Partial packing of PHABr showing a sheet of coplanar PHA + molecular cations with the pattern of hydrogen bonds involving the water solvent molecules, Figure S5: Crystal packing viewed along the normal to (a–c) direction showing sheets of PHA+ molecular cations piled up in the (a–c) direction. H-bonds are drawn as cyan lines. Hanging contacts are drawn as red lines, Figure S6: Left: Two-dimensional fingerprint plot of PHABr. The pair of sharp spikes at about di + de = 2.1 Å symmetrically disposed with respect to the diagonal are due to O…H/H…O interactions. The green area at di + de = 3.6 Å is diagnostic of π···π stacking interactions due to C/C interactions. Right: The flat shape of the molecule is evident in the curvedness (A) surface and in the shape index (B) surfaces. The pattern of red and blue triangles on the same region of the shape index surface is characteristic of molecules overlapping with π···π stacking, Figure S7: SCD profile of the lipid bilayer: a) oleic chains and b) palmitic chains of POPC. The order parameters of the lipid alone at pH = 7.0 is shown in blue. The SCD of the lipid chains at pH = 7.0 and pH = 9.0 upon interaction with the PHA+ probe are shown in orange and grey respectively, Figure S8: Area per lipid in Å2 for pure POPC membrane (control) and POPC lipids in the presence of PHA + at pH = 7.0 and PHA ± at pH = 9.0, Figure S9: Mass spectrum of the precursor 5-(4-formyl-3-hydroxyphenoxy)-N,N,N-trimethylpentan-1-aminium bromide in 50% CH3CN solution, performed using a Q-TOF premier instrument (Waters, Milford, MA, USA) equipped by an electrospray ion source and a hybrid quadrupole-time of flight analyser, acquired in positive ion mode, over the 100–1000 m/z range, Figure S10: Mass spectrum of the sensor PHA + in 50% CH3CN solution, performed using a Q-TOF premier instrument (Waters, Milford, MA, USA) equipped by an electrospray ion source and a hybrid quadrupole-time of flight analyser, acquired in positive ion mode, over the 100–1000 m/z range, Table S1: Crystal data and structure refinement details for PHABr. Table S2. Selected bond lengths (Å) and angles (°) for PHABr with e.s.d.’s in parentheses, Table S3. Hydrogen bonding geometry for PHABr (e.s.d.’s in parentheses).
