# Supplementary material for: An Amphiphilic Pyridinoyl-hydrazone Probe for Colorimetric and Fluorescence pH Sensing
Source: Molecules. 2019 Oct 24;24(21):3833. doi: 10.3390/molecules24213833 (PMC6864485; doi:10.3390/molecules24213833)
Supplement: Supplementary file 1 [file molecules-24-03833-s001.pdf]

An amphiphilic pyridinoyl-hydrazone probe for colorimetric and fluorescence pH sensing

Rosita Diana[a], Barbara Panunzi[a,*], Angela Tuzi[b], Stefano Piotto[c], Simona Concilio[d], and Ugo Caruso[b]

[a] Department of Agriculture, University of Napoli Federico II, via Università 100, 80055 Portici NA, Italy

[b] Department of Chemical Sciences, University of Napoli Federico II, via Cintia, 80126 Napoli, Italy

[c] Department of Pharmacy, University of Salerno, via Giovanni Paolo II 132, 84084 Fisciano SA, Italy

[d] Department of Industrial Engineering, University of Salerno, via Giovanni Paolo II 132, 84084 Fisciano SA, Italy

*corresponding author: barbara.panunzi@unina.it

Supplementary Materials

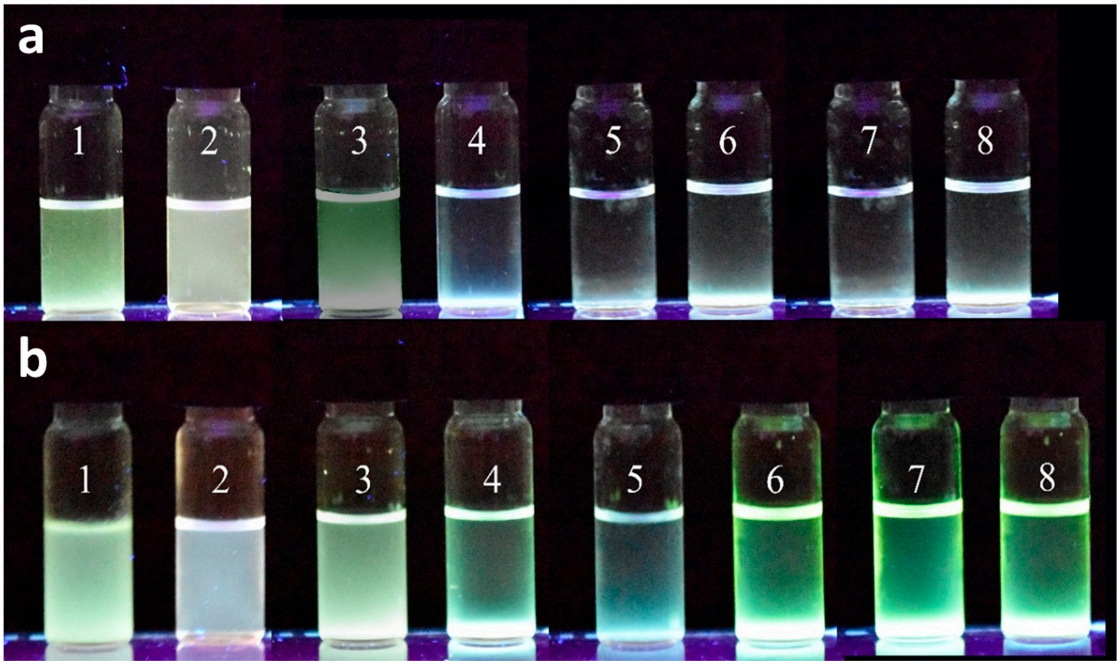

Figure S1. PHABr sensor in 100 μM solutions of different solvents (solvents 1-8, in increasing polarity order): 1 = chloroform, 2 = dioxane, 3 = acetone, 4 = ethanol, 5 = acetonitrile, 6 = DMF, 7 = DMSO and 8 = distilled water, (a) before and (b) after addition of NaOH up to pH=8.0.

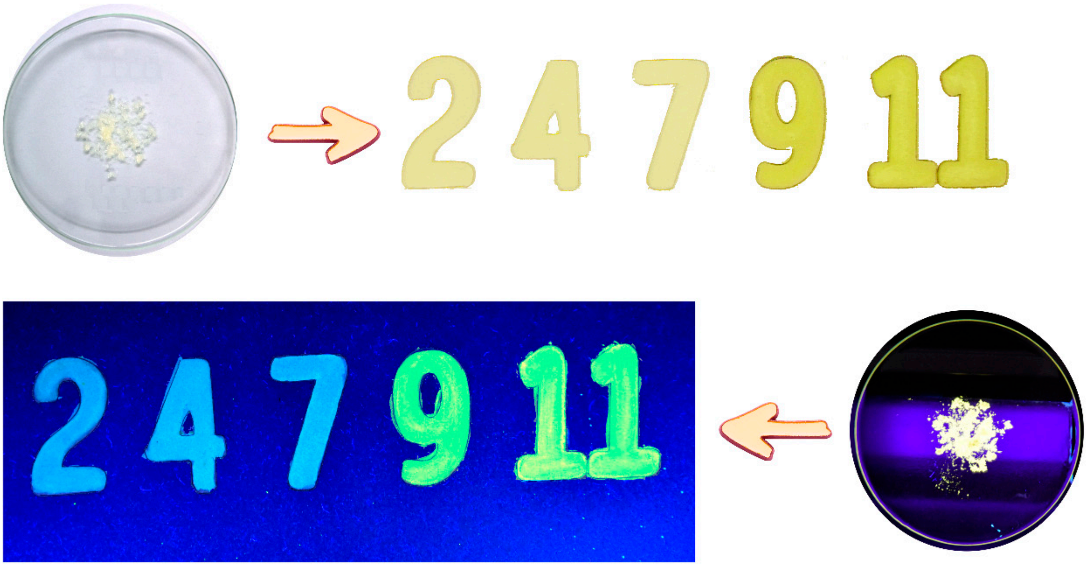

Figure. S2 Colour of probe **PHABr** as a crystalline solid in natural light (Petri dish on the left) and resting on the UV lamp at 365 nm (the same Petri dish on the right). Colour of common laboratory paper soaked in the probe at pH 2.0, 4.0, 7.0, 9.0, and 11.0 photographed at visible light (above) and under UV lamp at 365 nm (down).

Table S1. Crystal data and structure refinement details for **PHABr**.

	**PHABr**
CCDC number	1939629
Empirical formula	$C_{21}H_{29}N_4O_3Br \cdot 4(H_2O)$
Formula weight	537.45
Temperature (K)	298(2)
Wavelength (Å)	0.71073
Crystal system (Å)	Monoclinic

Space group	$P2_1/c$
a (Å)	8.654(2)
b (Å)	25.531(5)
c (Å)	14.551(2)
α (°)	90
β (°)	125.684(18)
γ (°)	90
Volume (Å^3)	2611.4(10)
Z	4
D_{calc} (Mg/m^3)	1.367
μ (mm^{-1})	1.619
F(000)	1128
Crystal size (mm)	0.30 x 0.25 x 0.08
θ range (°)	2.348 to 24.992
Reflections collected / unique	9447 / 4349 [R(int) = 0.0461]
Refinement method	Full-matrix least-squares on F ^{2}
Data / restraints / parameters	4349 / 1 / 331
Goodness-of-fit on F ^{2}	1.039
Final *R* indices [I>2sigma(I)]	*R1* = 0.05550, *wR2* = 0.1602
R indices (all data)	*R1* = 0.0859, *wR2* = 0.19170
Largest diff. peak and hole (eA^{-3})	0.471 and -1.090

Table S2. Selected bond lengths (Å) and angles (°) for **PHABr** with e.s.d.'s in parentheses.

C6-O1	1.221(5)
C7-N3	1.277(5)
C9-O2	1.361(5)
N2-N3	1.382(5)
N1-C5-C6	118.1(4)
C6-N2-N3	117.5(4)
C7-N3-N2	117.2(4)
N1-C5-C6-N2	-3.9(5)
N3-C7-C8-C13	-178.5(4)
C12-C11-O3-C14	178.8(4)

Table S3. Hydrogen bonding geometry for **PHABr** (e.s.d.'s in parentheses).

D–H···A	d(D–H) Å	d(H···A) Å	d(D···A) Å	<(D–H···A) °
C2-H2...O4W^{i}	0.93	2.53	3.300(7)	140.7
C7-H7...O3W^{ii}	0.93	2.61	3.386(6)	140.7

C19-H19A...Br1	0.96	3.07	3.911(5)	146.4
C19-H19B...O1^{iii}	0.96	2.40	3.269(7)	150.0
C19-H19C...O4W^{iv}	0.96	2.64	3.570(8)	163.2
C21-H21A...Br1^{v}	0.96	3.00	3.952(5)	169.9
C21-H21B...O1^{iii}	0.96	2.38	3.252(6)	150.7
N2-H2N...O3W^{ii}	0.87(5)	2.14(5)	2.994(6)	167(4)
O2-H2O...N3	0.73(5)	1.97(6)	2.594(5)	143(6)
O1W-H1A...Br1	0.71(6)	2.66(6)	3.367(4)	173(7)
O1W-H1B...O2W^{vi}	0.80(2)	1.95(2)	2.743(6)	173(6)
O2W-H2A...O2iii	0.82(8)	2.04(8)	2.843(6)	164(7)
O2W-H2B...Br1	0.66(7)	2.69(7)	3.336(4)	166(9)
O3W-H3A...Br1	0.70(6)	2.74(6)	3.427(4)	168(7)
O3W-H3B...O1W^{vii}	0.78(6)	2.12(6)	2.872(6)	164(7)
O4W-H4A...O1W^{vii}	0.95(7)	1.87(7)	2.817(7)	174(7)
O4W-H4B...Br1	1.02(7)	2.35(8)	3.366(5)	173(6)

Symmetry code: *i* = -x+2, y+1/2, -z+3/2; *ii* = -x+3, -y, -z+2; *iii* = x+2, y, z+1; *iv* = x-1, -y-1/2, z-1/2; *v* = x-1, y, z; *vi* = x, -y-1/2, z-1/2; *vii* = x+1, -y-1/2, z+1/2.

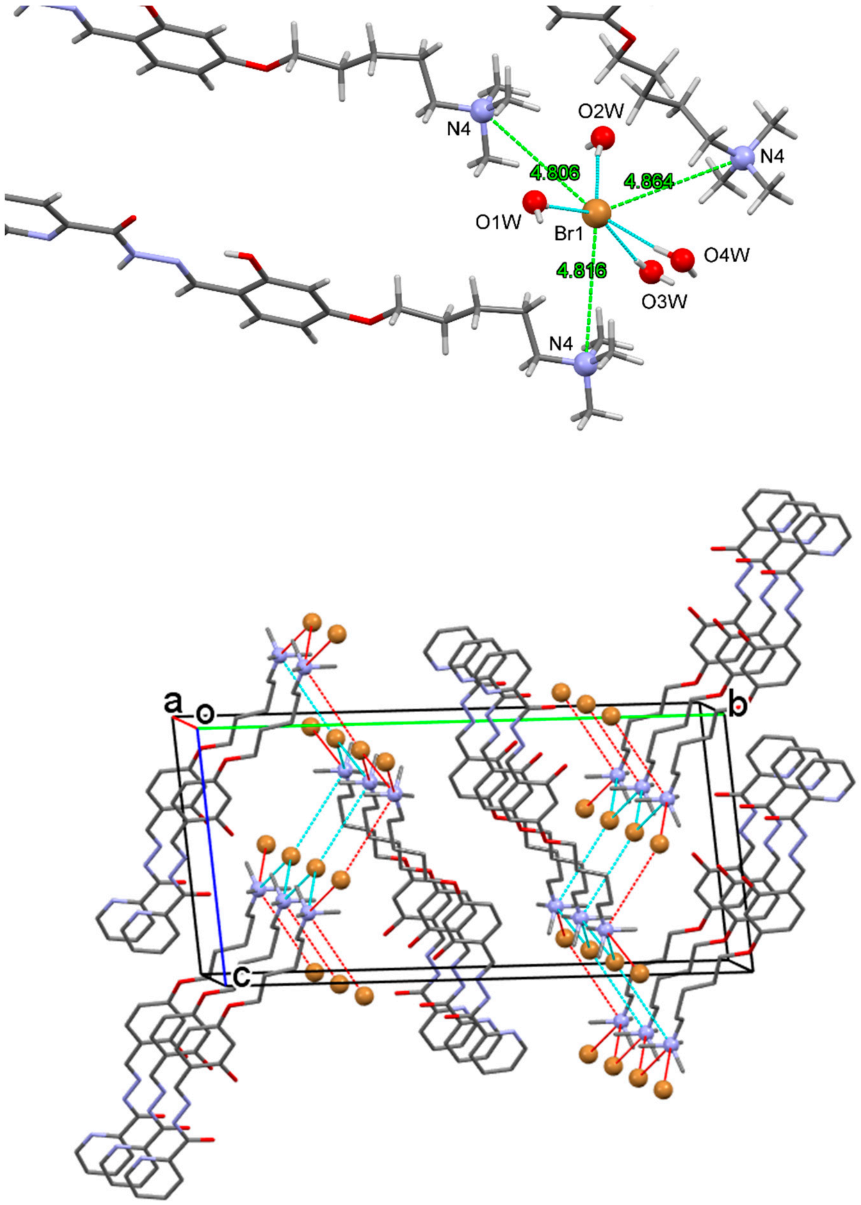

Figure S3. Up**:** Br^- anion surroundings with four H-bonded water molecules contained in the first coordination sphere and three -$N(CH_3)_3$ groups in the second coordination sphere at N4…Br1 distance of about 4.8 Å. Down: Pattern of anions and cations in the crystal packing. Water molecules and H atoms are not reported for clarity. Bromine and N(trimethylammonium) atoms are drawn as ball-and-stick stile, all the other atoms as wireframe style.

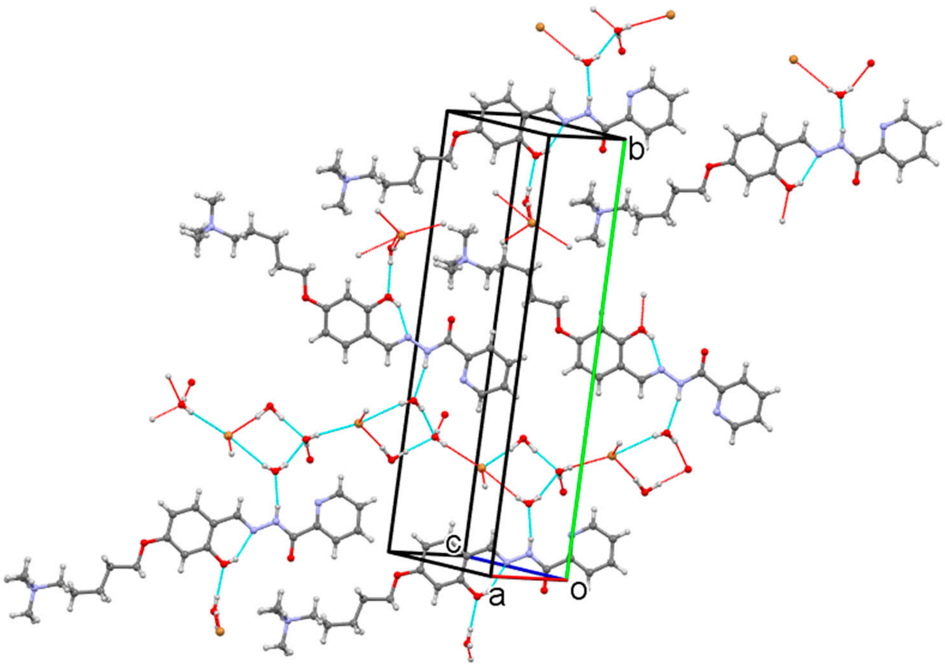

Figure S4. Partial packing of **PHABr** showing a sheet of coplanar **PHA**$^+$ molecular cations with the pattern of hydrogen bonds involving the water solvent molecules.

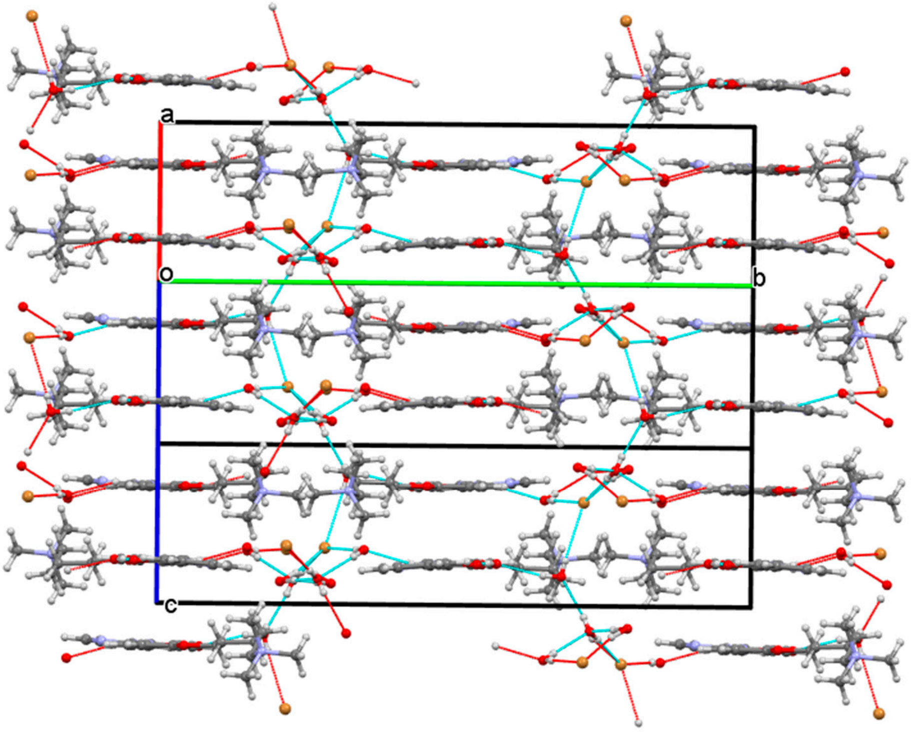

Figure S5. Crystal packing viewed along the normal to (**a**-**c**) direction showing sheets of **PHA**$^+$ molecular cations piled up in the (**a**-**c**) direction. H-bonds are drawn as cyan lines. Hanging contacts are drawn as red lines.

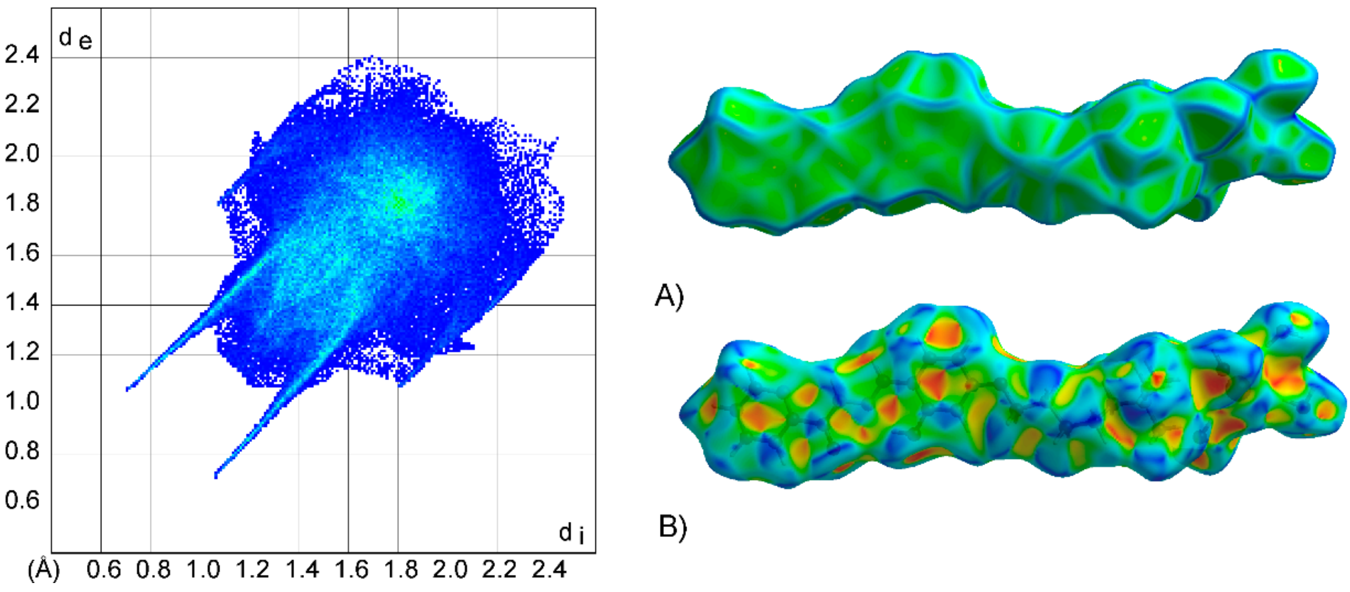

Figure S6. Left: Two-dimensional fingerprint plot of **PHABr**. The pair of sharp spikes at about $d_i + d_e = 2.1$ Å symmetrically disposed with respect to the diagonal are due to O…H/H…O interactions. The green area at $d_i + d_e = 3.6$ Å is diagnostic of π···π stacking interactions due to C/C interactions. Right: The flat shape of the molecule is evident in the curvedness (A) surface and in the shape index (B) surfaces. The pattern of red and blue triangles on the same region of the shape index surface is characteristic of molecules overlapping with π···π stacking.

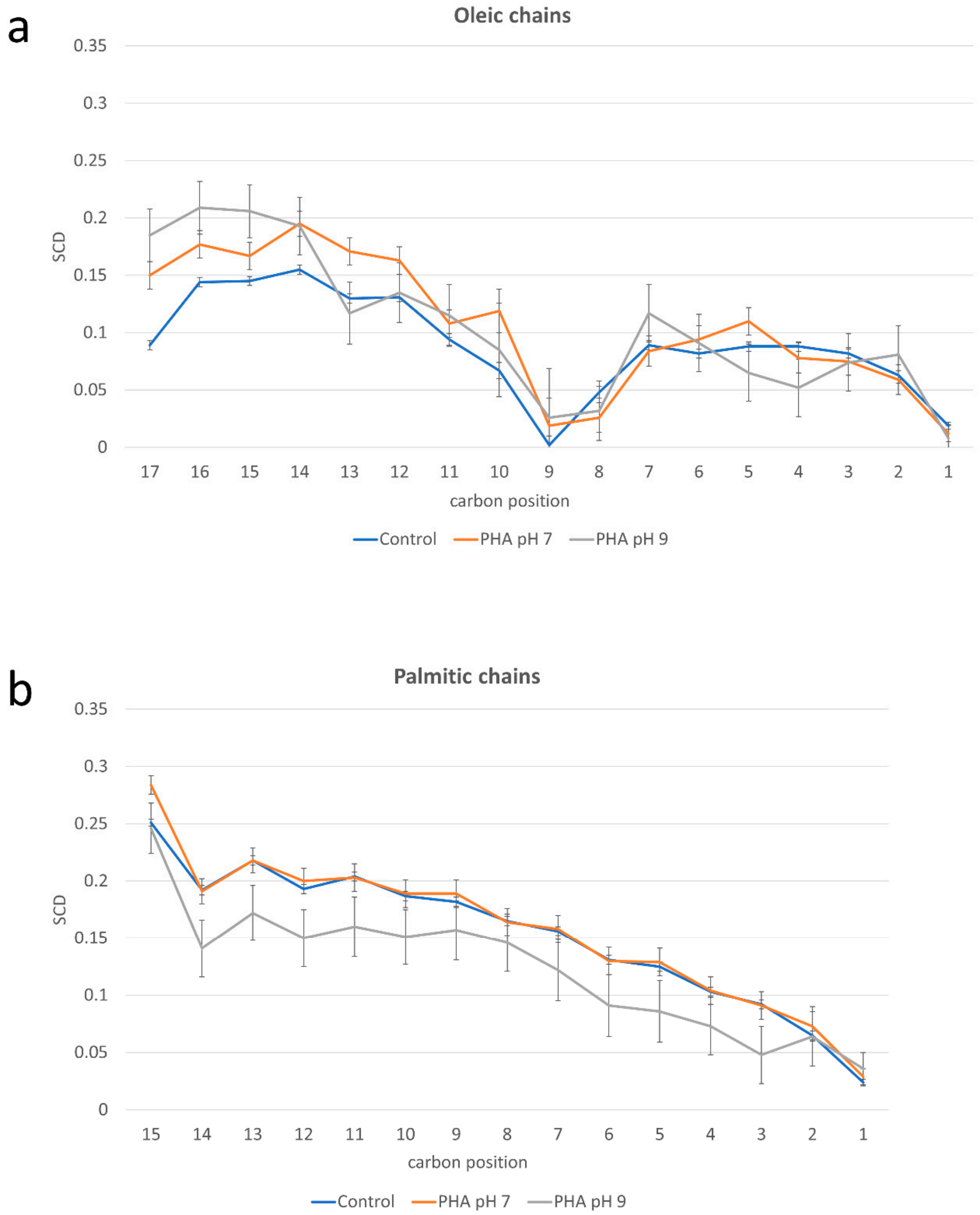

Figure S7. SCD profile of the lipid bilayer: a) oleic chains and b) palmitic chains of POPC. The order parameters of the lipid alone at pH=7.0 is shown in blue. The SCD of the lipid chains at pH=7.0 and pH=9.0 upon interaction with the **PHA$^+$** probe are shown in orange and grey respectively.

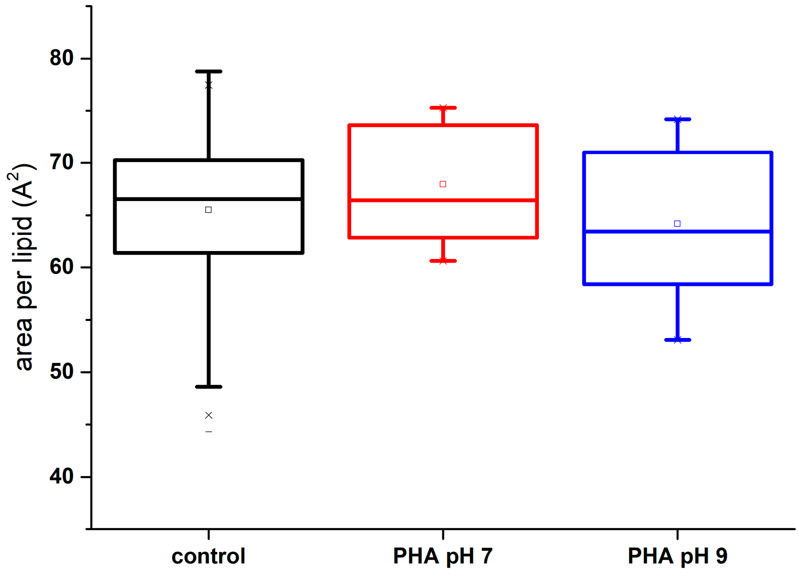

Figure S8. Area per lipid in Å^2 for pure POPC membrane (control) and POPC lipids in the presence of **PHA**$^+$ at pH=7.0 and **PHA**$^{\pm}$ at pH=9.0.

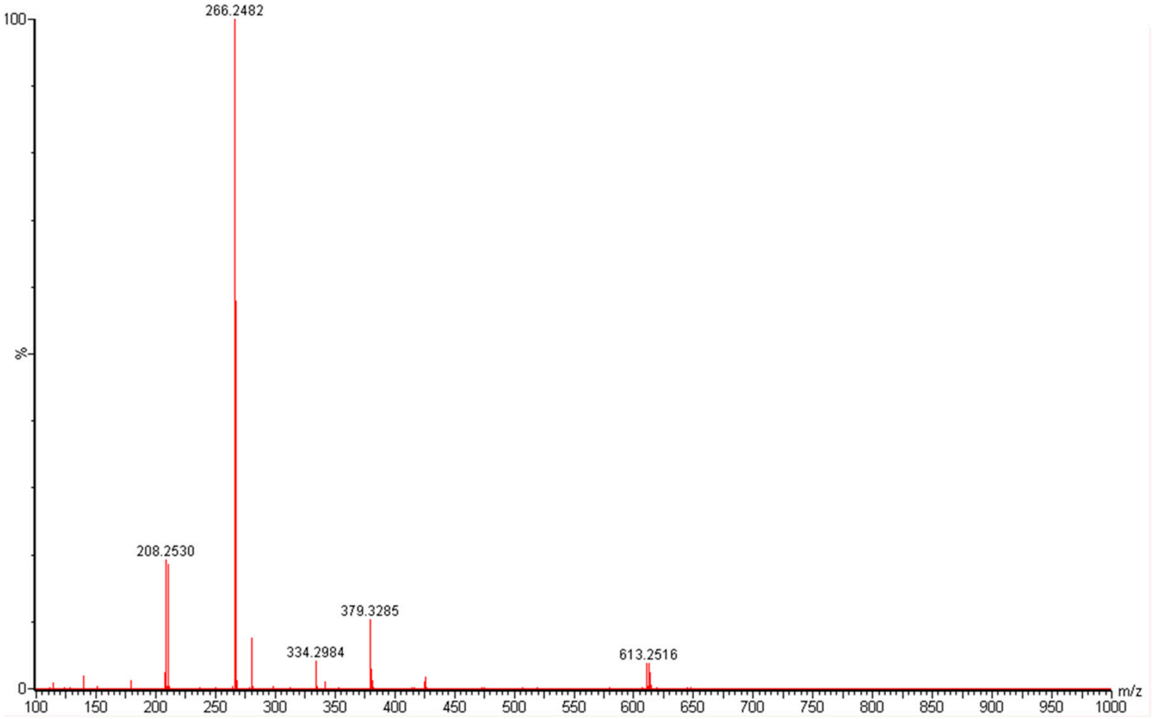

Figure S9. Mass spectrum of the precursor 5-(4-formyl-3-hydroxyphenoxy)-N,N,N-trimethylpentan-1-aminium bromide in 50% CH_3CN solution, performed using a Q-TOF premier instrument (Waters, Milford, MA, USA) equipped by an electrospray ion source and a hybrid quadrupole-time of flight analyser, acquired in positive ion mode, over the 100-1000 m/z range.

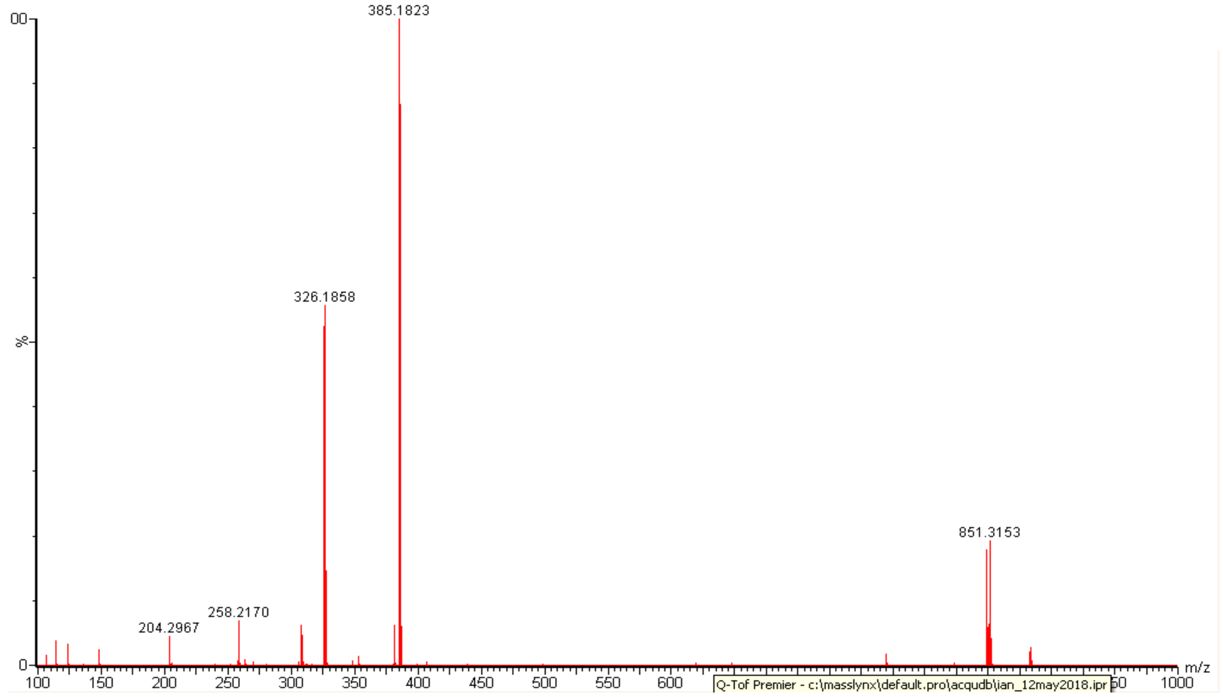

Figure S10. Mass spectrum of the sensor **PHA⁺** in 50% CH_3CN solution, performed using a Q-TOF premier instrument (Waters, Milford, MA, USA) equipped by an electrospray ion source and a hybrid quadrupole-time of flight analyser, acquired in positive ion mode, over the 100-1000 m/z range.
